# Perspective: A Framework to Screen Pediatric and Adolescent Hematopoietic Cellular Therapy Patients for Organ Dysfunction: Time for a Multi-Disciplinary and Longitudinal Approach

**DOI:** 10.3389/fonc.2021.622630

**Published:** 2021-02-24

**Authors:** Ali H. Ahmad, Kris M. Mahadeo

**Affiliations:** ^1^ Pediatric Critical Care Medicine, University of Texas at MD Anderson Cancer Center, Houston, TX, United States; ^2^ Pediatric Stem Cell Transplantation and Cellular Therapy and CARTOX Program, University of Texas at MD Anderson Cancer Center, Houston, TX, United States

**Keywords:** pediatric stem cell transplantation, pediatric critical care, multiple organ dysfunction, pediatric critical care illness severity scores, pediatric multi-organ dysfunction syndrome

## Abstract

Hematopoietic Cell Transplantation (HCT) is a potentially curative therapy for children and adolescent/young adults (AYA) with high-risk malignancies as well as some non-malignant genetic diseases. However, HCT may be associated with endotheliopathies and/or organ dysfunction that may progress to pediatric multi-organ dysfunction syndrome (pMODS) and require critical care intervention. Discipline specific scoring systems may be used to characterize individual organ dysfunction, but the extent to which they are used to prospectively monitor HCT patients with mild dysfunction is unknown. Further, separate scoring systems may be used to define risk of mortality and inform prognostication among those who require critical care support. Our understanding of the epidemiology, risk factors, morbidity, mortality, required monitoring, optimal prevention strategies and appropriate management of children undergoing HCT who develop organ dysfunction, endotheliopathies and/or progress to pMODS is poor. Discipline-specific registries and clinical studies have described improving outcomes for children undergoing HCT, including those who require critical care support; however, longitudinal studies/prospective registries that capture common data elements among HCT patients with and without organ dysfunction, endotheliopathies and pMODS are needed to facilitate inter-disciplinary collaboration and optimally characterize the risk profiles, define screening and prophylaxis regimens and mitigate toxicity.

## Introduction

Advancements in transplantation and supportive care have led to marked improvement in outcomes of hematopoietic cell transplant (HCT) patients who require pediatric intensive care unit (PICU) support (1). Endothelial cell activation post-HCT may trigger complications such as capillary leak syndrome, engraftment syndrome, transplant-associated microangiopathy (TMA), diffuse alveolar hemorrhage (DAH), idiopathic pneumonia syndrome (IPS) and sinusoidal obstructive syndrome (SOS) (2). Clinical manifestations of these disorders may overlap with each other as well as with other post-HCT complications such as graft-versus-host-disease (GVHD) (2). Thus, the individual contribution of these disorders to organ dysfunction and subsequent failure have been poorly defined. While some studies have characterized the clinical progression of HCT patients with pediatric multi-organ dysfunction syndrome (pMODS) once in the PICU, there is a paucity of information regarding predisposition, incidence, spectrum of severity and overall and functional outcomes of HCT patients with organ dysfunction who do not require critical care support (3–9).

Improved characterization of post-HCT complications that do not escalate to require PICU admission may facilitate more precise risk stratification. Use of well-defined screening and diagnostic criteria may allow for a clear-cut comparison among HCT patients who do and do not require PICU admission. Further, application of discipline specific organ screening criteria may improve inter-disciplinary collaboration to follow the progression of asymptomatic and/or severe organ dysfunction over time. Accurate recognition of predisposing factors and prompt diagnosis of endotheliopathies and organ dysfunction may mitigate the progression to pMODS and need for critical care support and/or improve survival following PICU admission (3–9).

Here, we review existing pediatric screening and diagnostic algorithms for organ toxicity and provide a framework for prospective comprehensive screening of pediatric-AYA patients undergoing HCT, which incorporates the use of internationally accepted discipline specific standard definitions.

### Overview of HCT and Associated Toxicities

HCT is a potentially curative treatment for children with malignant and non-malignant diseases (10).Patients receive a preparative regimen (chemo and/or radiotherapy) that may vary in intensity based on disease indications and/or host factors that is followed by infusion of either autologous (patient's own) or allogeneic (donor's) stem cells. Administration of the preparative regimen prior to HCT, may result in tissue damage, associated cytokine release and activation of endothelial cells. Translocation of endotoxins across damaged mucosa, infections, white blood cell recovery and engraftment (in particular, allogeneic donor cell engraftment) all contribute to a pro-inflammatory cytokine milieu which may exacerbate endothelial dysfunction (11). Infection is a significant cause of HCT morbidity and mortality, especially in the early neutropenic phase. Graft-versus-host-disease (GVHD) is a complication of allogeneic HCT, where a donor's allo-reactive T-lymphocytes view the recipient's healthy cells as foreign and damage them. Poorly controlled infections, GVHD and post-HCT endotheliopathies have the potential to evolve into multi-organ dysfunction syndrome (MODS). MODS is defined as the presence of altered organ function in an acutely ill patient such that homeostasis cannot be maintained without intervention (12). It is characterized by the concurrent dysfunction of two or more organs or systems including respiratory, cardiovascular, hematological, neurological, gastrointestinal, hepatic and renal (9, 13–15). An overview of HCT and possible toxicities and important considerations are presented in **Table 1**.

**Table 1:** Transplant Associated Complications and Considerations.

Timepoint	Possible Complications	Considerations
Pre-HCT	Confirm eligible malignant or non-malignant disease present Confirm suitable donor availability Organ Assessment: Tailored based on prior clinical history and specific pre HCT diagnosis and planned preparative regimen Infectious disease screening Psycho-social screening: emotional support; post-discharge care	Ensure appropriate informed consent/assent discussion. Transfusion informed consent Consider critical care introduction to patient/family. Consider open discussion around goals of care, quality of life considerations and advanced directives.
Preparative Regimen	Nausea/vomiting; hair loss; pancytopenia; transaminitis; acute kidney injury; mucositis; seizures; syndrome of inappropriate antidiuretic hormone secretion (SIADH)	Establish transfusion parameters for anemia and thrombocytopenia. Prophylactic anti-microbials; high-vigilance sepsis protocols; appropriate isolation. Prophylactic anti-emetics and pain medications and oral care for mucositis. Assess for enteral feeds and/or total parenteral nutrition. Monitor for fluid overload with hyper-hydration protocols. Seizure precautions (anti-epileptics as appropriate, for example with all patients receiving busulfan and/or patients with sickle cell disease receiving calcineurin inhibitors) Psycho-social support
HCT Infusion	Infusion reactions	Pre-medication with anti-pyretic, anti-histamines and/or corticosteroids. Anaphylaxis precautions.
4–6 weeks post-HCT	Pancytopenia Mucositis Infection Sinusoidal obstructive syndrome (SOS) Transplant-associated microangiopathy (TMA) Diffuse alveolar hemorrhage (DAH) Idiopathic pneumonia syndrome (IPS) Engraftment syndrome Graft-versus-host-disease (GVHD) Delirium Posterior reversible encephalopathy syndrome (PRES) Cerebrovascular accident (CVA) Seizures Acute kidney injury (AKI) Hepatic Impairment Hemorrhagic cystitis	Monitor for transfusion reactions Growth factors when indicated Monitor for viral reactivation. Sepsis may present without a fever Refractory thrombocytopenia may be an early indicator of SOS New infiltrate on chest imaging with hypoxia and drop in hemoglobin may indicate DAH Delirium may be iatrogenic (review medications and interactions) Refractory hypertension may be associated with PRES AKI may be subtle at presentation and/or related to endotheliopathy, multi-organ dysfunction and/or medication related Hepatic impairment may be medication related; high vigilance for SOS
Critical Care Admission	Usually respiratory indication Other indications may include: shock, need for renal replacement therapy, CVA, uncontrolled seizures	Recommend co-management between ICU and HCT and symptom management teams Ensure cross-discipline comprehensive understanding of the patient Careful and frequent medication reconciliation and renal dosing when appropriate Maintain infection precautions (patients usually profoundly immune compromised) Monitor for GVHD Discuss any planned use of steroids between HCT and ICU teams (may deplete circulating lymphocytes, obscure GVHD) Therapeutic drug monitoring: Ensure central line lumens for calcineurin inhibitors are not interchanged with blood draw lines. Review goals of care at specified intervals and as clinically indicated

### Post-HCT Endotheliopathies

Post-HCT endotheliopathies include capillary leak syndrome (CLS), engraftment syndrome (ES), transplant-associated microangiopathy (TMA), diffuse alveolar hemorrhage (DAH), idiopathic pneumonia syndrome (IPS) and sinusoidal obstructive syndrome (SOS). ^2^ CLS is characterized by the loss of intravascular fluids into interstitial spaces and is dominated by sudden weight gain, generalized edema unresponsive to diuresis and hypotension, which may eventually lead to cardiovascular shock with respiratory and pre‐renal insufficiency (16). ES is characterized by fever, skin rash, pulmonary edema, weight gain, liver and renal dysfunction in addition to encephalopathy and it occurs at the time of neutrophil recovery after HCT (17, 18). TMA may be diagnosed using published diagnostic criteria that includes (1) lactate dehydrogenase (LDH) elevated above the upper limit of normal for age (2) de novo thrombocytopenia with a platelet count <50 × 10^9^/L or a ≥50% decrease in the platelet count (3) de novo anemia with a hemoglobin below the lower limit of normal or anemia requiring transfusion support (4) microangiopathic changes defined as the presence of schistocytes in the peripheral blood or histologic evidence of microangiopathy on a tissue specimen and (5) absence of a coagulopathy and a negative Coombs test. Laboratory criteria must occur concurrently, and criteria 1 to 4 should be documented on at least two consecutive tests to be classified as positive. ADAMTS13 activity should be measured to exclude a diagnosis of thrombotic thrombocytopenic purpura (19, 20). SOS, formerly referred to as hepatic veno-occlussive disease (VOD), may be diagnosed by modified EBMT Pediatric Criteria which require two or more of the following: unexplained consumptive and transfusion-refractory thrombocytopenia; otherwise unexplained weight gain on three consecutive days despite the use of diuretics, or a weight gain of more than 5% above baseline value within 72 h; bilirubin increasing from baseline on three consecutive days, or bilirubin 2 mg/dl or more within 72; hepatomegaly (best if supported by imaging) above baseline value; and ascites (best if supported by imaging) above baseline (21). DAH is a devastating non-infectious complication following HCT defined as a syndrome of hypoxia, dyspnea, infiltrates on chest radiograph, and progressively bloodier bronchoalveolar lavage or the presence of hemosiderin-laden macrophages on microscopy (22, 23). IPS is defined as the presence of multi-lobar infiltrates by chest radiograph or computed tomography scan, need for supplemental oxygenation with declining pulse oximetry and no identifiable pulmonary infection (24).

## Organ Dysfunction Syndromes Post-HCT

### Respiratory

Pulmonary complications post-HCT are common, with respiratory failure the leading cause of PICU admission among this population, and a significant source of non-relapse mortality. Respiratory complications post-HCT can be categorized into infectious and non-infectious, with imperfect diagnostic strategies that too often rely upon the presence of a constellation of overlapping clinical symptoms and diagnoses of exclusion. The Pediatric Acute Lung Injury Consensus Conference (PALICC) established criteria for diagnosis and severity grading of pediatric acute respiratory distress syndrome (PARDS) in children (25). Among children post-HCT who develop respiratory illness, there is a very high risk of developing severe PARDS irrespective of the underlying cause of pulmonary dysfunction, with a mortality rate of 40% to 60%. Emerging data suggest that longer duration of respiratory distress, increased use of non-invasive ventilation, and/or supplemental use of oxygen prior to intubation are associated with higher mortality for these children (4, 26).

Respiratory insufficiency in children post-HCT may result from a variety of causes such as post-HCT endotheliopathies, acute fluid overload and/or infection. The true incidence of respiratory insufficiency in pediatric HCT patients encompassing those who do and do not progress to failure is unknown. Emergency response systems such as the pediatric early warning system (PEWS) monitor indicators, such as heart rate, respiratory rate, systolic blood pressure, capillary refill time, work of breathing, oxygen therapy, and transcutaneous oxygen saturation (27). Among pediatric oncology and HCT patients, critical deterioration (defined as unplanned PICU transfer requiring life-sustaining interventions within 12 h) is preceded by a long duration of abnormal vital signs, making it potentially preventable through prompt recognition (28). Understanding the relative contributions of post-HCT complications to development of respiratory failure may further improve risk mitigation strategies.

### Cardiovascular

Arrhythmias and cardiac dysfunction are not insignificant post-HCT complications in children. Known risk factors include exposure to anthracycline-based chemotherapeutic regimens and/or prior thoracic or total body irradiation. Prospective data that assesses the impact of degree of acute fluid overload and/or blood pressure variations, incidence and significance of arrhythmias, pericardial effusions as well as the role of potential biomarkers such as brain natriuretic peptide (BNP) and galectin-3, with regard to post-HCT outcomes may inform future monitoring and management strategies (29, 30). The New York Heart Association (NYHA) Heart Failure classification is not applicable to most of the pediatric population. The Ross Heart Failure classification, developed to assess severity in infants and subsequently modified to apply to all pediatric ages, provides a numeric score comparable with the NYHA classification for adults (31).While the general principles of heart failure management may be similar to adults, there is a compelling need for larger and higher-quality studies regarding cardiac failure in children undergoing HCT to provide a more robust evidence base (32).

### Renal

Known risk factors for acute kidney injury (AKI) in the pediatric HCT population include allogeneic HCT, sinusoidal obstructive syndrome (SOS), use of nephrotoxic medications thrombotic microangiopathy (TMA), prior history of AKI, decreased baseline glomerular filtration rate (GFR), total body irradiation, and myeloablative chemotherapy conditioning regimens (33–42). DiCarlo and Alexander described the evolution of organ dysfunction in pediatric HCT as a result of a cytokine driven process, which may first manifest as fluid accumulation (38).Acute fluid overload above specific thresholds may be associated with high mortality rates in pediatric HCT patients who require PICU admission and detection of AKI often occurs well after the window for potentially successful mitigation strategies have passed (43, 44). The true incidence and precise impact of acute fluid overload among pediatric HCT patients who do not require PICU admission remains poorly characterized and clinical and physiological studies to date, demonstrate that the ideal fluid strategy in AKI has not been developed. Serum creatinine is insufficiently sensitive to detect “renal angina” and has been especially problematic for clinical research in pediatric AKI. The Kidney Disease Improving Global Outcomes (KDIGO) definition and staging of AKI was recently adopted into the severity staging for pediatric SOS and may be used to guide clinical care based on consensus of pediatric nephrology experts (21, 45). The renal angina index (RAI), determined by a composite of vasopressor use, invasive mechanical ventilation, percent fluid overload, and estimated creatinine clearance, may improve prediction of subsequent severe AKI (KDIGO Stage 2 to 3) in critically ill children when compared with an increase in serum creatinine alone (46, 47). These classification systems require prospective validation among pediatric HCT patients which may inform optimal strategies for AKI mitigation, fluid management (restrictive versus permissive), diuresis, thresholds for initiation of renal replacement therapy and placement of fluid drains.

### Hepatic

Pediatric patients undergoing HCT are at risk for liver dysfunction from exacerbation of pre-existing co-morbidities such as prior infection, iron overload, SOS and/or hepatotoxic drugs, preparative regimens as well as allo-reactivity. No reliable tools exist to predict hepatic injury and overall survival or death in patients with pediatric acute liver failure. Existing liver failure scoring systems include the Child-Pugh score, Model for End-Stage Liver Disease and Pediatric End-Stage Liver Disease score, and the Liver Injury Unit score (48–51). These scoring systems incorporate ascites, encephalopathy, bilirubin kinetics, and coagulopathy and together resemble elements included in the current severity grading for sinusoidal obstructive syndrome in pediatric patients now routinely used among pediatric HCT patients (21). Vigilance for SOS in pediatric HCT patients based on current diagnostic criteria may facilitate early recognition of hepatic injury and inform future prophylaxis and treatment strategies. Sheer wave elastography ultrasound studies may represent a promising strategy to augment emerging vigilance protocols (52). Indeed, prospective data regarding the true incidence and spectrum of hepatic injury in pediatric HCT, including progression to pMODS are needed.

### Neurologic

Neurological complications post-HCT may results from infection, metabolic derangements (due to medication or organ dysfunction), anatomical or metabolic abnormalities associated with the underlying diagnosis, or cerebrovascular events and are a significant cause of HCT-related mortality (53). Presenting symptoms may include delirium, seizure, encephalopathy, altered mental status, headache, or focal neurological signs. While little is known regarding true predisposition, a history of a neurological event prior to HCT has been associated with an increased risk of neurological complications post-HCT. Posterior reversible encephalopathy syndrome (PRES) may be one of the most common neurological complications in HCT patients that prompts transfer to the PICU. PRES may present with headache, acute mental status changes, visual changes including cortical blindness, and seizures, generally in association with an acute rise in blood pressure. Signal abnormalities on FLAIR imaging in the posterior regions of the brain, reflective of vasogenic edema may be present on magnetic resonance imaging (MRI). Cerebral vascular dysregulation, in response to elevated blood pressure or to endothelial activation, may cause vasogenic edema, commonly in the parieto-occipital regions, but may be found elsewhere. PRES has most commonly been associated with the administration of calcineurin inhibitors, sirolimus and dexamethasone. Usually reversible, PRES may be associated with severe morbidity and mortality if unrecognized. CNS infections in HCT patients are relatively rare but associated with potentially severe sequelae (54–56). For example, progressive multifocal leukoencephalopathy (PML) is a demyelinating disease due to JC virus infection that is found late (months to years) post-HCT. Optimal screening algorithms for neurologic toxicity post-HCT may reduce progression to severe complications. Screening and other mitigation strategies for endotheliopathies, GVHD and infection may result in prompt recognition and appropriate management. Early clinical signs and symptoms of neurological complications may be subtle. The Cornell Assessment of Pediatric Delirium (CAPD) provides a validated screening tool for delirium, which may be an early symptom associated with neurological complications (57, 58).The CAPD scoring system is a familiar tool used on pediatric HCT units since it has been incorporated into diagnostic and severity grading criteria for immune-effector cell associated neurotoxicity (ICANS) as well as SOS severity grading (21, 59).

### MODS

As delineated above, pediatric patients undergoing HCT are at risk for organ dysfunction that may progress to pMODS. Over three decades, several scoring methods have emerged to define pediatric MODS, including the Pediatric Logistic Organ Dysfunction (PELOD) Score, Pediatric Multiple Organ Dysfunction Score (pMODS), and the pediatric Sequential Organ Failure Assessment (pSOFA) score (60–62). Also during this time, several studies reported pediatric MODS incidence rates ranging between 6% and 57% in the general PICU population, across all diagnoses (63–69). As mentioned previously, HCT is a well-described risk factor for pediatric MODS (5, 7, 62, 70).Additionally some studies suggest that the pediatric HCT subpopulation may display more severe and/or unique manifestations of organ dysfunction syndromes, including acute respiratory failure, sepsis and delirium, compared to the general pediatric population (71–73).

## Discussion

Currently, no ideal scoring system exists to appropriately define organ dysfunction and risk of severity in pediatric HCT patients. Many of the existing clinical algorithms identify patients at risk for rapid clinical deterioration once admitted to the PICU but do not pre-emptively identify patients with mild abnormalities at risk for pMODS; none are specific to HCT patients, where consideration of the cumulative effect of mild dysfunction in multiple organs may be important. A pre-transplant HCT Comorbidity Index score >3 as commonly used in adult patients, has been associated with inferior survival of patients undergoing allogeneic HCT for some non-malignant diseases (74). However, 69% of pediatric patients in these cohorts may have a score of 0 prior to transplantation and the index does not provide dynamic and prospective considerations following HCT (74).

Binary descriptors of normal function versus dysfunction do not reflect cumulative decrements in organ function which may indicate progressive increase in mortality. Herein, as shown in **Table 2**, we provide a framework for prospective toxicity scoring tool that highlights progressive organ and immune dysfunction that can be used throughout an HCT admission (including PICU). Use of cross-discipline scoring systems that have been adapted to the expected ranges for pediatric HCT patients, is meant to facilitate a broader understanding of the patient’s clinical status among inter-disciplinary clinical specialties. Incorporation of discipline specific elements such as KDIGO and CAPD with standard HCT variables may foster a broader inter-disciplinary approach to the pediatric HCT patient. We have also included notation to reflect patients who have organ dysfunction and are receiving end-of-life-care. Goals of care may change during a patient’s clinical course and it is important that respective disciplines are aware. In the future, prospective validation of such a tool may help delineate cumulative toxicity scores and/or thresholds that warrant specific interventions. We hope that this tool stimulates inter-disciplinary conversation and prompts an effort for validation of this or a similar scoring system. The scores for individual observation points are meant to highlight severity of that system only (which is not always appreciated outside of specific disciplines). For example, KDIGO monitoring may not be routinely performed on HCT units and its significance may not be promptly appreciated by an HCT clinical team. Similarly, the impact of absolute lymphocyte count or higher grade graft-versus-host-disease may not be promptly recognized by a critical care team. The impact of cumulative toxicity scores among multiple observation points will require further investigation. In the interim, we hope that tools such as this, will promote a more comprehensive approach to the HCT patient.

**Table 2:** Possible Screening for pMODS for Pediatric and Adolescent-Young Adult (AYA) HCT Patients^ab^.

Score+	0	1	2	3	4	5
**Respiratory** ^c^ Oxygen support on the HCT floors PaO_2_/FiO_2_ (P/F) ratio SpO_2_/FiO_2_ (P/F) ratio	Room air	Blow-by oxygen and ≥400 ≥292	Nasal Cannula or 300-399 264-291	NIV or 200-299 221-263	MV and/or 100-199 with respiratory support 148-220 with respiratory support	MV and/or <100 with respiratory support <148 with respiratory support
**CV (MAP by age group, mmHg)^d^** < 1 month 1-11 months 12-23 months 24-59 months 60-143 months 144-215 months >216 months or Vasoactive infusion, μg/kg/min	≥ 46 ≥ 55 ≥ 60 ≥ 62 ≥ 65 ≥ 67 ≥ 70	< 46 < 55 < 60 < 62 < 65 < 67 < 70	Dopamine hydrochloride < 5 or dobutamine hydrochloride (any)	Dopamine hydrochloride 5-9.9 or epinephrine < 0.1 or norepinephrine bitartrate ≤ 0.1	Dopamine hydrochloride 10-14.9 or epinephrine 0.1-0.2 or norepinephrine bitartrate 0.1-0.2	Dopamine hydrochloride > 15 or epinephrine > 0.2 or norepinephrine bitartrate > 0.2
**Renal** KDIGO AKI Criteria Patients must have one of the following 1. Increase in baseline Serum creatinine (bSCr) ≥ 0.3 mg/dL within 48 hrs 2. Increase in bSCr ≥ 1.5x baseline that is known or presumed to have occurred within past 7 d 3. Urine volume < 0.5 mL/kg/hr for 6 hr	Baseline (No AKI)			KDIGO 1 1.5-1.9 x bSCr or Cr increase > 0.3 mg/dL or Urine volume < 0.5 mL/kg/hr for 6-12 hours	KDIGO 2 2-2.9 x bSCr or Urine volume < 0.5 mL/kg/hr for >12 hours	KDIGO 3 >3 x bSCr or Cr > 4 mg/dL or Initiation of RRT or Urine volume < 0.5 mL/kg/hr for > 24 hours or Anuria> 12 hours
**Renal** Weight gain – after diuretics	Baseline	2-5%	>5-10%	>10%	Persistent rise >10%	RRT
**Hepatic** Total Bilirubin	Baseline			≥ 2	Doubles in 48h	Doubles in 24h
**Hematologic** INR or Refractory Thrombocytopenia	<1.2	1.2 -1.5 < 3 days	>1.5-1.9 3-7 days	≥2	Need replacement of factors > 7 days	Active Bleeding
**CNS** CAPD^e^	Baseline or <9	Initial increase from baseline, but < 9	Sequential increase from baseline, but < 9	≥ 9	Sequential increase > 9	≥ 9 and/or recent/active CVA, PRES, or seizures
**Immune Reconstitution** ^f^ ANC ALC Acute GVHD (75) Active infection	>1500/mm^3^ >1500/mm^3^ None None	>1000-1500/mm^3^ >1000-1500/mm^3^ Stage 1 H/o clinically significant infection	500-1000/mm^3^ >800 -1000/mm^3^ Stage 2 Active controlled	< 500/mm^3^ 500-800/mm^3^ Stage 3 Active uncontrolled	<200/mm^3^ <500/mm^3^ Stage 4 Multiple active/uncontrolled infections	<100/mm^3^ <200/mm^3^ Stage 4 Multiple active/uncontrolled infections

a. May be performed weekly and if clinically significant deterioration. Use the worst value in preceding 24-hour period for each variable b. If concern for pMODS, recommend further screening for endotheliopathies such as CLS, ES, TMA, DAH, IPS, and/or SOS. c. P/F ratio to be used when arterial blood gas is available. Otherwise, use S/F ratio. d. MAP = (1/3 x SBP) + (2/3 x DBP) e. CAPD change from baseline should also be taken into consideration when using CAPD score. e. ANC: absolute neutrophil count [white blood cell count (k/uL) x (%neutrophils+ bands) x 10 f. ALC: absolute lymphocyte count [white blood cell count (k/uL) x (% lymphocytes) x 10 + patients receiving end of life care may be delineated with an organ score and “E” (example 4E); this designation is intended to retain awareness of specific goals of care and explicitly state rationale when invasive organ support interventions are not initiated. f. Assign the highest score if any 1 criteria is met in this category.

The use of clinical scoring systems that encompass discipline specific tools may facilitate cross-talk, promote inter-disciplinary research and advance our understanding of the complex interactions involved in pMODS. Given the rarity of childhood diseases and enrollment on clinical studies as compared to adults, it is essential that collaborations aimed at collecting broad clinical data to compliment biospecimen collection are developed in pediatric HCT (76). The Center for International Blood and Marrow Transplant Research (CIBMTR) and Virtual Pediatric Systems capture discipline specific data in disparate registries. Attempts to merge these registries to advance our understanding of pediatric HCT patients who require PICU care have been an important step forward to improve our working knowledge of the factors influencing the progression of critical illness in pediatric allogeneic HCT patients (77). However, even these efforts are limited by the limitations of current data fields which may not capture detailed organ specific information, in particular among Pediatric HCT patients who do not require PICU admission or who do become critically ill but proceed to palliative care in lieu of escalation of care.

Attention to changes in clinical variables that inform organ assessments among pediatric HCT and PICU patients such as used in SOS severity and pSOFA scoring may normalize the use of common discipline specific variables among inter-disciplinary teams (21, 64). In a validation study by Matics and Sanchez-Pinto, the pSOFA score was adapted to pediatrics by modifying age-dependent cardiovascular and renal variables of the original SOFA score using validated cutoffs from the PELOD-2 scoring system, and also by expanding the respiratory subscore to include the SpO2/FiO2 (S/F) ratio as an alternative surrogate for PaO2/FiO2 (P/F) ratio to assess lung injury (64). The derivation of S/F ratio to impute for P/F ratio in the respiratory subscore has been validated in other studies (65). Pediatric SOS severity scores includes common CTCAE organ toxicity assessments with adjustments for pediatric ages when appropriate (21). This includes use of discipline specific variables such as CAPD and KDIGO organ assessments. Given the overlap between pSOFA and SOS scoring, we depict in **Table 2**, our proposed screening for all pediatric HCT patients at baseline, serially (perhaps weekly during inpatient transplant hospitalization) and during sentinel events (including development of endotheliopathy, PICU admission, transition to end-of-life care). Capture of raw data from such screening into a multi-center prospective registry as planned by PALISI-Network centers should serve as an invaluable resource for inter-disciplinary collaboration.

Advancements in cellular therapy and regenerative medicine may allow for therapeutic interventions that may mitigate organ toxicity. For example, mesenchymal stem cells (MSC) may be used to control heart failure in patients with anthracycline induced cardiomyopathy (78).Umbilical cord blood and MSC infusions are currently under investigation in the treatment of some pediatric brain injuries, including autism (79). Rapid advancement in potential therapeutics must be matched by innovative approaches to optimize available clinical data in small pediatric cohorts. Innovative approaches that integrate a “big data” inter-disciplinary platform for pediatric HCT patients are desperately needed. Herein, we propose a framework to screen for organ toxicity and express support for a broad pediatric HCT registry that is linked to a bio-specimen repository.

## Data Availability Statement

The original contributions presented in the study are included in the article/supplementary material. Further inquiries can be directed to the corresponding author.

## Author Contributions

AA—First authorship, initial draft and revisions, and primary literature review. KM—Last authorship, revisions and new content, and secondary literature review. All authors contributed to the article and approved the submitted version.

## Conflict of Interest

The authors declare that the research was conducted in the absence of any commercial or financial relationships that could be construed as a potential conflict of interest.
