# Corrigendum: Perspective: A Framework to Screen Pediatric and Adolescent Hematopoietic Cellular Therapy Patients for Organ Dysfunction: Time for a Multi-Disciplinary and Longitudinal Approach

**DOI:** 10.3389/fonc.2021.679526

**Published:** 2021-04-23

**Authors:** Ali H. Ahmad, Kris M. Mahadeo

**Affiliations:** ^1^ Pediatric Critical Care Medicine, University of Texas at MD Anderson Cancer Center, Houston, TX, United States; ^2^ Pediatric Stem Cell Transplantation and Cellular Therapy and CARTOX Program, University of Texas at MD Anderson Cancer Center, Houston, TX, United States

**Keywords:** pediatric stem cell transplantation, pediatric critical care, multiple organ dysfunction, pediatric critical care illness severity scores, pediatric multi-organ dysfunction syndrome

In the original article, there was a mistake in **Table 2**. Possible Screening for pMODS for Pediatric and Adolescent-Young Adult (AYA) HCT Patients as published.

**Table 2:** Possible Screening for pMODS for Pediatric and Adolescent-Young Adult (AYA) HCT Patients^ab^.

Score+	0	1	2	3	4	5
**Respiratory** ^c^ Oxygen support on the HCT floors PaO_2_/FiO_2_ (P/F) ratio SpO_2_/FiO_2_ (P/F) ratio	Room air	Blow-by oxygen and ≥400 ≥292	Nasal Cannula or 300-399 264-291	NIV or 200-299 221-263	MV and/or 100-199 with respiratory support 148-220 with respiratory support	MV and/or <100 with respiratory support <148 with respiratory support
**CV (MAP by age group, mmHg)^d^** < 1 month 1-11 months 12-23 months 24-59 months 60-143 months 144-215 months >216 months or Vasoactive infusion, μg/kg/min	≥ 46 ≥ 55 ≥ 60 ≥ 62 ≥ 65 ≥ 67 ≥ 70	< 46 < 55 < 60 < 62 < 65 < 67 < 70	Dopamine hydrochloride < 5 or dobutamine hydrochloride (any)	Dopamine hydrochloride 5-9.9 or epinephrine < 0.1 or norepinephrine bitartrate ≤ 0.1	Dopamine hydrochloride 10-14.9 or epinephrine 0.1-0.2 or norepinephrine bitartrate 0.1-0.2	Dopamine hydrochloride > 15 or epinephrine > 0.2 or norepinephrine bitartrate > 0.2
**Renal** KDIGO AKI Criteria Patients must have one of the following 1. Increase in baseline Serum creatinine (bSCr) ≥ 0.3 mg/dL within 48 hrs 2. Increase in bSCr ≥ 1.5x baseline that is known or presumed to have occurred within past 7 d 3. Urine volume < 0.5 mL/kg/hr for 6 hr	Baseline (No AKI)			KDIGO 1 1.5-1.9 x bSCr or Cr increase > 0.3 mg/dL or Urine volume < 0.5 mL/kg/hr for 6-12 hours	KDIGO 2 2-2.9 x bSCr or Urine volume < 0.5 mL/kg/hr for >12 hours	KDIGO 3 >3 x bSCr or Cr > 4 mg/dL or Initiation of RRT or Urine volume < 0.5 mL/kg/hr for > 24 hours or Anuria> 12 hours
**Renal** Weight gain – after diuretics	Baseline	2-5%	>5-10%	>10%	Persistent rise >10%	RRT
**Hepatic** Total Bilirubin	Baseline			≥ 2	Doubles in 48h	Doubles in 24h
**Hematologic** INR or Refractory Thrombocytopenia	<1.2	1.2 -1.5 < 3 days	>1.5-1.9 3-7 days	≥2	Need replacement of factors > 7 days	Active Bleeding
**CNS** CAPD^e^	Baseline or <9	Initial increase from baseline, but < 9	Sequential increase from baseline, but < 9	≥ 9	Sequential increase > 9	≥ 9 and/or recent/active CVA, PRES, or seizures
**Immune Reconstitution** ^f^ ANC ALC Acute GVHD (75) Active infection	>1500/mm^3^ >1500/mm^3^ None None	>1000-1500/mm^3^ >1000-1500/mm^3^ Stage 1 H/o clinically significant infection	500-1000/mm^3^ >800 -1000/mm^3^ Stage 2 Active controlled	< 500/mm^3^ 500-800/mm^3^ Stage 3 Active uncontrolled	<200/mm^3^ <500/mm^3^ Stage 4 Multiple active/uncontrolled infections	<100/mm^3^ <200/mm^3^ Stage 4 Multiple active/uncontrolled infections

a. May be performed weekly and if clinically significant deterioration. Use the worst value in preceding 24-hour period for each variable b. If concern for pMODS, recommend further screening for endotheliopathies such as CLS, ES, TMA, DAH, IPS, and/or SOS. c. P/F ratio to be used when arterial blood gas is available. Otherwise, use S/F ratio. d. MAP = (1/3 x SBP) + (2/3 x DBP) e. CAPD change from baseline should also be taken into consideration when using CAPD score. e. ANC: absolute neutrophil count [white blood cell count (k/uL) x (%neutrophils+ bands) x 10 f. ALC: absolute lymphocyte count [white blood cell count (k/uL) x (% lymphocytes) x 10 + patients receiving end of life care may be delineated with an organ score and “E” (example 4E); this designation is intended to retain awareness of specific goals of care and explicitly state rationale when invasive organ support interventions are not initiated. f. Assign the highest score if any 1 criteria is met in this category.

The following in **Table 2** has been corrected:

Respiratory: Score 0 and Score 2; CV: Score 2; Renal: Score 4; Hematologic: Score 0, Score 1, Score 2, and Score 3; CNS: Score 0 and Score 4; Immune Reconstitution: Score 1 and Score 2 and Footnote f.

The corrected *Table 2. Possible Screening for pMODS for Pediatric and Adolescent-Young Adult (AYA) HCT Patients* appears below.

The authors apologize for this error and state that this does not change the scientific conclusions of the article in any way. The original article has been updated.
